# Legal medicine implications in fibrinolytic therapy of acute ischemic stroke

**DOI:** 10.1186/s12910-019-0412-8

**Published:** 2019-10-14

**Authors:** Monica Sabau, Simona Bungau, Camelia Liana Buhas, Gheorghe Carp, Lucia-Georgeta Daina, Claudia Teodora Judea-Pusta, Bogdan Adrian Buhas, Claudia Maria Jurca, Cristian Marius Daina, Delia Mirela Tit

**Affiliations:** 1 County Clinical Emergency Hospital, Oradea, Romania; 2 Faculty of Medicine and Pharmacy, Department of Psycho-Neurosciences and Rehabilitation, University of Oradea, Oradea, Romania; 3 Faculty of Medicine and Pharmacy, Department of Pharmacy, University of Oradea, Oradea, Bihor Romania; 4 Faculty of Medicine and Pharmacy, Department of Morphological Disciplines, University of Oradea, 50 Clujului St., 410060 Oradea, Bihor Romania; 5 Bihor County Forensic Service, 50 Clujului St, 410060 Oradea, Bihor Romania; 6 Faculty of Medicine and Pharmacy, Department of Surgical Disciplines, University of Oradea, Oradea, Romania; 7 Municipal Clinical Hospital Cluj-Napoca, Cluj-Napoca, Romania; 8 Faculty of Medicine and Pharmacy, Department of Preclinical Disciplines, University of Oradea, Oradea, Romania; 9 Department of Genetics, Municipal Clinical Hospital, Dr. Gavril Curteanu, Oradea, Romania

**Keywords:** Stroke thrombolysis, Medical negligence, Prevention, Medico-legal issues in stroke

## Abstract

**Background:**

Before the advent of fibrinolytic therapy as a gold standard method of care for cases of acute ischemic stroke in Romania, issues regarding legal medicine aspects involved in this area of medical expertise were already presented and, in the majority of cases, the doctors seem to be unprepared for these situations.

**Main text:**

The present research illustrates some of the cases in which these aspects were involved, that adressed a clinical center having 6 years of professional experience in the application of fibrinolytic treatment for stroke. The following cases report either situations in which the afore mentioned therapy was not rightfully administrated or legal aspects regarding the obtainment of informed consent.

**Conclusion:**

Obtaining informed consent is a mandatory procedure, which takes time, to the detriment of application of fibrinolytic treatment.

## Background

### Fibrinolytic treatment

Thrombolysis represents without doubt an important step in the treatment of acute ischemic stroke / cerebral vascular accidents (CVA). Before the advent of fibrinolytic therapy, the treatment of stroke resided in reality only as a nihilistic conviction. In the cerebral area affected by lack of oxygen supply [1, 2], downstream of the arterial occlusion point, an infarction zone appears - ischemic necrosis of the cerebral parenchima. Around it, due to the presence of collateral circulation (its existence depending on multiple factors and quite fragile in nature), the penumbra zone can be found.

The first historical attempts in cerebral revascularization date back in the years 1955–1960 with the use of human or bovine thrombolyzines or streptokinase [3]. Due to the fact that bleeding resulted as an important complication of the procedure (10 out of 73 patients died in the first study), the treatment option was eventually abandoned; thrombolysis continued to be performed but only in the setting of an acute myocardial infarction and only to patients not presenting any signs of cerebral lesions.

In the years 1980th, numerous studies were re-performed with the administration of thrombolytics through intravenous (i.v.) or intraarterial (i.a.) paths leading to promising results.

Every case was investigated using angiography. The most important aspect, frenquently omitted in literature, is the fact that during the same period of time new methods for patients’ evaluation and follow up were developed and it was clearly proved that the dimensions of the cerebral infarcted area and patients’ clinical evolution are directly dependent upon the vascular occlusion [4].

After the years 1990, randomized studies appeared, with the use of recombinant tissue plasminogen activator (rtPA) compared with placebo. In these specific studies angiographies were not performed, patients were selected only on the basis of computer tomography evaluations (CT). NINDS National Institute of Neurological Disorders and Stroke (NNDS) presented the first study, their promising results being published in 1995 [5]. The study proved the efficacy of treatment with rtPA 0.9 mg/kg in patients with acute CVA in the first 3 h starting from the appearance of symptoms. Haemorrhagic conversions were 10 times more frequent than in the cases of patients receiving placebo but only a few of these transformations were symptomatic and responsible for the aggravation of the condition and death of the patients.

In most of the cases patients’ general status amelioration was remarkable with significant improvement of neurological deficit. In the following year FDA aproved the use of Alteplase in acute CVA within the first three hours from the moment of symptoms appearance.

Numerous other studies followed: ECASS I, ECASS II, ECASS III; ATLANTIS A, ATLANTIS B, IST-3 trial, as well as numerous re-interpretations of statistical results, which in the end validated the data upon the treatment benefits with alteplase and widened the therapeutical window [6–11]. Repeated evaluations and reviews [12–14] demonstrate the efficiency and underline the safety of thrombolytic therapy.

The last review elicits how *“Trials testing rtPA (n=7012) showed a significant reduction in death or dependency with treatment <6 hours (OR, 0.84; 95% CI, 0.77–0.93) with significant heterogeneity between trials. The rtPA treatment <3 hours was more beneficial (OR, 0.65; 95% CI, 0.54–0.80), without heterogeneity”* [14].

The benefits of the administration of thrombolytic agents reside in the fact that they lyse the thrombus determining the vascular occlusion. Revascularization gives the possibility of recovery for the remaining viable nervous tissue and due to this, it limits the expansion of the infarcted area and thus the grade of neurological deficiency.

The main drawback of this therapy consists in the narrow therapeutic window allowed for administration (initially set at 3 h, currently widened to 4.5 h). In this short interval of time, the patient has to be diagnosed, transported to a specialized unit, investigated and the therapy has to be applied.

### Implementing fibrynolitic therapy

Nevertheless, following all the favourable reviews and considering the lack of a valuable therapeutic alternative, the implementation of fibrinolytic treatment still remained a challenge in the whole world. In 2004, eight years after the approval of rtPA, Profesor Caplan concludes that doctors and medical centers respond slowly and difficultly to the call of fibrinolytic treatment use. At the time only 1–2% of patients with stroke were administered rtPA even though many more were eligible for treatment [4].

In modern years, in the USA and western states of Europe, important progress was obtained with the creation of networks connecting stroke centers with specialized equipment, trained personnel and sustained activity. In Romania and most of Eastern Europe countries the situation is still questionable. Doctors, hospitals and authorities respond slowly to change. Most of them are not yet ready to give an adequate medical assistance to a patient suffering of acute CVA.

In Romania, after a pilot project unveiled the mechanism and modality of action in fibrinolysis treatment in cases of stroke, the efforts of the Romanian Neurology Society board managed to lay the foundation of a national program known as “CVA Priority Acute Action”. The program involved many hospitals that met the necessary conditions and in which doctors were able and desired to contribute to the attainment of this treatment.

The protocol used for the application of the treatment is extremely clear, in concordance with the European and local guidelines, furthermore, being updated several times. There are no and there were no differences between the Romanian protocol and the European guidelines. The latest therapeutic protocol for thrombolytic and endovascular treatment in acute ischemic stroke implemented nationwide was developed in 2018, according to some documents [15–17].

There are a lot of reasons that make the implementation of this treatment difficult: besides the problematic activity of specialists’ coordination due to the limited time window available for treatment and the patient’s follow up being quite complicated, the most important factor to consider resides in the concern of physicians for the occurrence of haemorrhagic complications, cases in which they could be legally pursued. Together with the widening of the therapeutic window, the aforementioned concern became even more evident.

Our research presents the problems that arise in front of the neurologist in the process of obtaining the patient’s consent with over-acute vascular accident and the negative way in which these circumstances are reflected over the interval of intervention/treatment. On the other hand, there are cases where families expressly require this type of treatment, but patients are out of the criteria: clinical or time.

## Main text

### Legal medicine implications in fibrinolytic treatment

Fibrinolytic therapy has emerged as a “standard gold method” for the treatment of stroke, although this type of treatment is at the beginning in Romania; prior to the occurrence of this therapy, aspects of legal medicine involved in this field of medical expertise have already been reported. Experience has shown in most cases that physicians do not seem to be prepared to cope with emergency situations, especially when they have to obtain the consent of the patient or his care giver, being constrained by time, the informational level of the population, observance of the legal issues, etc.

Since March 2012 when this type of treatment was started, fibrinolysis was used in 200 cases of acute CVA at Oradea Emergency County Hospital, Romania. This accounts for approximately 3% of the total acute CVA cases. The percentage reported in our study (3%) is lower than those reported by similar studies (about 6% in India or less than 7% in the US) [18, 19].

In Romania, the introduction of thrombolysis is an important service development for acute stroke services, which was implemented a few years later than in Western countries. At the time of reporting these cases, there were only 7 hospitals in the country where thrombolysis was performed. The lack of centers in each county and the poor road infrastructure in the country determine the increase of the time necessary to get to the hospital. Therefore, the most common cause that impedes venous thrombolysis is exceeding the 4.5 h therapeutic window. The causes are multiple and are associated with the pre-hospital stage; among the most frequent are: the patient’s ignorance of stroke symptoms and implicitly the unawareness of the onset of the disease, as well as the inefficient management of the patient with stroke by the intervention teams.

In 2018, only 14 hospitals in Romania performed this service. Basically, over 75% of the counties did not benefit from a single adequately equipped hospital, where the thrombolysis procedure would be applied for the cases of ischemic strokes, while the other counties benefited from a single hospital. This critical situation has determined the Ministry of Health to take measures to extend the program nationwide. As a result, since the beginning of 2019, more than 95% of the counties have at least one hospital where thrombolysis is performed. By Order of the Minister 170/2019 the protocol for interventional treatment of patients with acute stroke was approved - a set of measures, procedures and directions regarding the care of the patient with acute stroke in the pre-hospital stage, in order to increase the efficiency of the emergency system, as well and in the hyperacute phase (within hospitals).

These measures, along with public education programs to quickly recognize the signs of stroke, and the training of intervention teams for effective management of the patient with stroke in the therapeutical time window for thrombolysis, aim to increase the number of patients receiving this treatment. Some of these cases that specifically involed legal medicine issues are further presented.

### Cases presentation

#### Case 1

Female patient, age 87, known with breast cancer, with multiple bone metastasis (vertebral, ribs, pelvis), with chronic atrial fibrillation (AF), without anticoagulant treatment, collapsed in her home and was found on the floor with motor deficit localized on the left side of her body. An ambulance was called to the scene and the patient arrived shortly at the emergency department. The emergency physician evaluated the patient and, although the time of onset of her neurological symptoms was unclear, he did not consult the neurologist for thrombolysis.

Neurological examination revealed: patient was conscious, temporo-spatially well oriented as well as self aware but couldn’t specify the moment of her collapse. The patient presented left hemiplegia, left hemianestesia, left homonymous hemianopsia, anosognosia. Close relatives and friends of the patient stated that she was last seen without neurological symptoms in the evening of the previous day. Thus, the moment of the neurological deficit appearance was still unknown, but the last moment when she was noticed without these signs places her well outside of the therapeutic window.

CT examination didn’t reveal any cerebral lesions (ASPECTS 10 points) and the laboratory analyses didn’t show any modification that could contraindicate thrombolysis treatment. In consequence, clinical examination, CT and laboratory analyses allow the patient to rtPA administration. Since the time span was unknown, the problem of fibrinolysis wasn’t even addressed. In these conditions, the physicians didn’t discuss with the patient nor with her relatives the possibility of treatment. Thus, the patient was admitted in the hospital, the diagnosis of acute CVA located in the area of the right middle cerebral artery was established, and treatment was implemented according to the protocol concerning strokes outside the range of therapeutic window. The evolution was unfavourable, her neurological status aggravated, coma was installed and complications arised: bronchopneumonia, numerous hydroelectric abnormalities, haemodynamic instability and at last death.

The family filed a complaint arguing that the fibrinolytic therapy was not administered even though the patient arrived at the hospital in less than two hours. According to them, the fact that the treatment was not administered led to the worsening of her clinical status and finally to her death. The complaint was directly addressed to the emergency physician, the neurologist and the hospital. Based on the data recorded in the medical file and the fact that the precise timing of the onset of symptomatology was unknown, the resolution of the case favored the hospital and doctors.

#### Case 2

Male patient, age 63, presented sudden onset right hemiparesis. The ambulance service was immediately activated, and he was transported to the hospital where the arrival of the patient, being still in therapeutical window for thrombolysis, was communicated. The patient was rapidly evaluated by the neurologist. The CT examination showed already signs of early cerebral infarcted areas because of which the patient was classified as ineligible for rtPA administration: ASPECTS score 5 points.

The patients’ relatives were informed about the existence of the procedure as well as about the benefits it can offer, so they insisted upon the administration of this treatment. The neurologist explained the reasons of his own refusal to administer the treatment: the extended lesion area and the cerebral infarction presence. In this specific case, the risk of haemorrhagic episode exceeding the benefits of the procedure, the neurologist completed the patient’s medical selection record and rtPA was not administered.

Nevertheless, the family’s desire for the administration of rtPA led to the filing of a written appeal towards the hospital management board in which it was specified the request for rtPA administration “upon one’s own responsibility”, and a second opinion to be given by another neurologist and radiologist was also required. As conclusion, the results yield by the imaging investigations excluded the patient from the possibility of administering fibrinolytic therapy.

After the patient was admitted, his clinical status worsened, and he developed a massive spontaneous haemorragic episode. He died after 7 days. The family did not file a complaint but remained sceptic regarding the correct medical approach to the matter.

It is important to specify that an eventual complaint filed by the patient’s relatives wouldn’t have had any legal claims due to the fact that the correctly filled medical records clearly prove that the patient was not eligible for the treatment.

A particularly troublesome issue for patients that could benefit from the fibrinolytic therapy is represented by the obtainment of the informed consent. This is due to the fact that in most cases they present neurological deficits that include aphasia which causes them the impossibility of expressing the consent for treatment. Some examples are described in the following cases.

#### Case 3

Female patient, age 65, brought from her home with right sided hemiplegia and aphasia with onset 2 h priorly. Urgent evaluation was performed in 40 min (160 min after the onset of the neurological deficit) and showed that the patient was eligible for fibrinolytic treatment. Because the patient couldn’t understand the spoken language and couldn’t reply, telephonic contact was established with her family who were on the way towards the hospital. They did not understand the situation and expressively announced that they preferred to arrive at the hospital in order to make a decision. The family eventually arrived at the hospital after the 4.5 h time window interval from the moment of neurological onset, thus the rtPA administration was not sufficeable anymore. The patient was discharged with severe neurological deficit, immobilized in bed and with complete aphasia.

#### Case 4

Female patient, age 67, brought to the hospital with right hemiplegia and aphasia, with 30–40 min after the onset of neurological symptoms. Urgent evaluation of the patient concluded that she was elibigle for fibrinolytic treatment. The decision was presented to the family in less than 90 min from the onset of symptoms. The patient was unable to sign the informed consent because she couldn’t understand spoken or written language, nor could she articulate verbally or by writing. The family decided not to sign the consent file for the administration of rtPA but agreed for the patient’s admission. The patient was eventually transferred to a local hospital, after 5 days in Oradea Emergency Clinical Hospital, with severe neurological deficit.

#### Case 5

Male patient, age 63, collapsed on the street and developed right sided hemiplegia and aphasia. He was brought to the emergency department and preparations for thrombolysis procedures were arranged. Doctors concluded that he was eligible for fibrinolysis treatment, but the patient was aphasic and couldn’t understand spoken language, nor could he articulate verbally. He was not registered in the electronic archive of the hospital with any illness that could represent a contraindication to the procedure.

A commission composed of one neurologist, two emergency physicians (the departament supervisor and the attending physician) was formed and decided to administer rtPA after analysing the risk-benefit ratio. The evolution was favourable, showing almost complete recovery from the neurological deficit and a small non-hemorrhagic brain lesion. The patient was discharged with slight neurologic deficit.

Another reason why obtaining informed consent in such cases is difficult, is the lack of understanding of the treatment and its benefits despite explaining them to the patients.

#### Case 6

Male patient, age 63, collapsed in a public place, he developed left hemiplegia and was brought to the emergency department. He was oriented in time and space, being aware of his hemiplegia. The initial evaluation showed that the patient was eligible for rtPA treatment. The neurologist explained to the patient the nature of his disease and that administering rtPA increased the chances of recovery, then asked him to sign the informed consent. The patient couldn’t read, as he was missing his glasses and despite having the document read out loud to him, refused to sign. He declared that he lived on his own, had no close family, never required treatment and did not believe drugs had any benefit. He agreed to the hospital admission, but not to the rtPA treatment. He was later discharged with motor deficit, being taken care of by the social services.

#### Case 7

Male patient, age 69, during his inpatient located in the cardiology department, due to his permanent atrial fibrillation, suddenly developed left hemiplegia. A neurologist was notified, and he performed an urgent evaluation 60 min after the onset of neurologic symptoms. The evaluation showed that the patient was eligible for administering rtPA. The patient refused fibrinolytic treatment and signed against it. He later discussed with his family and changed his mind. As he was still in the therapeutic window, he was administered rtPA after 170 min since the onset of the motor deficit. Immediately after administering rtPA there was a significant clinical improvement, but the follow-up CT showed petechial hemorrhagic lesions. The patient was discharged and sent to a rehabilitation clinic.

## Discussions

Rules of deontology and medical ethics regulate the professional liability of the physician in front of the medical professional comunity and in relation to the patient. The deontological laws regulate the legal liability of the doctor in relation to the patient (common law) and society (criminal law) [20, 21]. Certainly, all of the aforementioned are included in the legislation and the deontological rules of the Romanian College of Physicians (in Romanian: Colegiului Medicilor din România) and will not be detailed in this study. The discussion will be focused on the interpretations of these cases from the point of view of forensic training and practical application [22].

The presented cases show the difficulties that physicians encounter when applying fibrinolytic therapy. The decisions made by physicians are questioned by the patients or by their relatives. From this point the distance towards filing an official complaint is very short. The relatives that filed a complaint against the terapautical approach in the presented case 1 knew that the administration of rtPA depends on a certain time window and they were also informed about the considerable benefits of the treatment. They considered that as a consequence of the refusal to administer the treatment, the patient lost a chance of recovery without taking into account the fact that it was not known when the symptoms occurred. Due to the fact that the patient was conscious, they assumed that she knew the circumstances of her accident. It is difficult to understand a clinical condition such as anosognosia for someone who is not familiar with the medical field.

Considering that the exact timing of symptomatology onset was unknown, neither the emergency nor the neurology physicians took into consideration fibrinolysis treatment. The refusal of administering the treatment was justified and the protocol was respected. The medical files were correctly filled, and as a result the complaint was solved by favouring the accused.

It is obvious that the correct completion of all medical records is essential, especially in the case of a complaint file; the documents come to support the reasons for the medical decision. The superficial or incomplete completion of the data and information required may raise suspicions about the correctness and rigor of the medical act itself.

In the American literature, there are numerous studies concerning the legal implications of rtPA use. The medical system is generally more exposed to legal conflicts and compensations can reach outstanding numbers. Some studies present specifically the importance of correct filling of medical documents [23–25]. Such bibliographic sources were not found in European literature, and even less in Romania. Bhatt et al. reveal which are the factors that protect and favourize the medical staff in the case of a complaint: adequate documentation of the contraindications/indications for treatment; discussion upon risk-benefit ratio in more than 50% cases; expert testimony; clear statement of the symptomatology onset; obtaining informed consent; existence and respect of the protocol of the hospital [23]. Factors that can determine filing a complaint are: delayed diagnosis, incorrect diagnosis, the lack of transportation of the patient in a center that administeres rtPA, lack of informed consent, delayed or incorrect patient evaluation, existence of a complication during the treatment [26].

The second case (that did not end up with a complaint filing) shows the importance of discussing with the patient’s relatives. Through his decision, the physician opposed the desires and hopes of the family. Surely the clinical decision was a correct one, based on arguments encomprised in the therapeutic protocol. It is clear that the medical procedures can not be performed at the request of the family, if the patient is outside of the therapeutic recommendation range. In the case when the patient/family requests a certain treatment, which the physician considers unuseful or contraindicated or nonethic, the physician is not obliged ethically or legally to perform it, concept well underlined by every rule involving a correct medical practice. In this case 2, the administration of rtPA would have caused, most likely, a massive haemorrhagic episode.

In accordance with the last statement, the patient did experience a massive spontaneous haemorrhagic conversion which caused her death, even without the administration of the fibrinolytic treatment. The most correct approach in this case is the demand of a second opinion, which was actually requested. It is also important to consider the problem of differentiating between malpraxis and maloccurence (i.e. unfavourable evolution). The unfavourable evolution of a patient with acute CVA is quite frequent. It is a sudden onset situation, frenquently there are underlying unknown medical conditions, or even a group of conditions, that need multiple treatments and the acute CVA episode can determine the appearance of complications. Public perception of these differences is vague due to the fact that there are no measurable or well-defined variables. Each patient has its own particularities.

Concerning the situation of rtPA administration, the physician can receive a complaint whether he doesn’t, or he does administer the fibrinolytic agent, and complications arise. It is well known that the risk of a haemorrhagic episode exists even with a careful and correct selection of the patient. Literature reviews illustrate that most of the complaints were filed because of lack of thrombolytic treatment administration [22–28]. The data collected from the Neurology Ward in Oradea Emergency Clinical County Hospital show the same conclusions.

In case of emergency interventions in acute stroke, a complaint is most frequently encountered in omission situations; this is because, from the legal point of view, an action that has not been successful, but which has been done for the benefit of the patient is less contentious than the lack of action that passively consumes the possible chances of recovery of the patient.

The last presented cases all had as a main issue the informed consent. Surely informed consent is invariably necessary for every medical procedure. It is mandatory for the patient to be informed of any treatment or medical treatment and obtaining informed consent at the time of admission to any hospital unit does not imply acceptance of any further investigations or treatments [25].

Case 3 shows a situation in which the possibility of administering the treatment is lost because of the delay in receiving the informed consent. Frequently, family members trust the doctor and ask him to perform what he believes is best for the patient. There are also cases in which the family desires to understand and discuss further details and request explanations. In our experience this situation is accountable for the delayed administration of rtPA. The patient’s evaluation is performed, the decision to administer the fibrinolytic treatment is established but the medical team has to await the arrival of the family members.

In the mentioned case, because of the awaiting for the family members, the time window for the treatment was surpassed, leading to the impossibility of rtPA administration. Unfortunately, there are many other similar cases. In Oradea Emergency Clinical County Hospital, the most frequent reason for delayed treatment administration or for the inability to administer appropriate treatment in a timely manner was and is the obtainment of informed consent.

Case 4 illustrates the refusal of the family to accept the treatment. The patient cannot elicit her point of view because her language capacities are deeply affected: she cannot articulate verbally, nor can she understand spoken language. The risk of haemorrhagic complications and a therapeutic failure, both mentioned in the form of the informed consent and explained by the physician, determined the family to refuse the treatment.

The therapeutic protocol for thrombolytic and endovascular treatment in acute ischemic stroke in Romania (valid at the time of this study) was approved by a Ministerial Order. This protocol stipulates the obligation to obtain informed consent for thrombolysis. There is only one exception, namely when the patient does not have the capacity to express his or her consent and/or there are no caregivers: *“The patient will be informed about fibrinolytic therapy and will sign a consent form. The consent form includes information on both fibrinolysis and on the possibility of intra-arterial thrombolysis or thrombectomy, initially or after i.v. thrombolysis. If he can not sign, the verbal agreement will be recorded in the presence of a witness. If the patient can not express his agreement, the family can sign the consent form. If there are no caregivers, and the patient is confused, aphasic or has altered state of consciousness, the doctor can make the decision for thrombolysis if all the inclusion and exclusion criteria are met, the fibrinolytic treatment being included in the Diagnostic and Treatment Guide for Ischemic Stroke with indication Class IA “.*

On the other hand, Law no. 46 of January 21, 2003 (patient’s rights law), with subsequent modifications and completions, stipulates in Article 15: *“If the patient requires emergency medical intervention, the consent of the legal representative is no longer necessary”*, and in Article 17: *“ … if the legal representative refuses treatment or a medical procedure and the physician considers it to be in the patient’s interest, the decision is rejected by an arbitration comission consisting of 3 doctors, for hospitalized patients, and 2 physicians for ambulatory patients*” [29]. Both regulations are compulsory, their non-compliance producing legal effects in the case of complaints formulated by the patient. Performing thrombolysis in acute ischemic stroke is a medical emergency, taking into account the narrow therapeutic window, but the specific protocol for this intervention provides only one exception from obtaining the informed consent - the one described above. Thus, the dilemma in therapeutic decision making appears. In the particular cases presented (3 and 4), the doctors decided to strictly follow the rtPA specific protocol, and not the emergency exceptions provided by the law or the direct benefit of the patient. Another type of physician’ approach to the exceptions provided by the law would have led to the administration of rtPA, including these two cases. It is more than obvious that a congruence or agreement between the law and the protocol would make it easier for the doctor to decide from both therapeutic and ethical points of view. The situations of emergency exception to informed consent in the case of rtPA administration in acute ischemic stroke described in the literature [30] do not fully coincide with the Romanian protocol, and implicit consent management is permitted if there are no easily accessible sources of consent. If it is established that direct consent can not be provided by the patient, and also to avoid death or severe impairment of the patient, when no other form of consent can be obtained in time, implied consent remains more relevant for candidates for i.v. treatment with rtPA. An exception to informed consent may be invoked by a physician, if he has proper legal grounds, but the physician’s decision to apply the treatment without the informed consent raises an important ethical issue, namely whether the quality of being a doctor is sufficient to make a moral decision on behalf of another person [31]. Whenever possible, it is imperative that the physician obtains a direct consent from the patient candidate for i.v. rtPA therapy, taking into account the moral difficulties inevitable in alternative forms of consensus [30].

Case 5 shows a patient whose decision-making capacity is affected by speech disorders. Family contact data were not known because the patient was alone, and the installation of neurological signs/deficits happened in a public place. The medical team has fully respected the ethical and legal principles in taking decisions as regards the patient’s specific case. The result was satisfying. But what would have happened if the results weren’t good? What if the treatment wouldn’t have been efficient or complications would have arisen? Of course, a complaint would have been in order, but the way the decision was made and the existence of a team of physicians who made that specific decision would have supported the correctness of the medical act. These cases refer to patients who have lost their decision-making capacity due to speech disorders in the context of stroke. The legislation states that, in these situations, the family or friends are the ones who have to give their consent. However, information given to a third person about a patient can generate problems related to the professional secrecy. What is the concept of friend? Who is defined as a friend?

Some emergency services require patients to agree to inform their family relatives of their state of health; this rule was established after some patients have complained of non-compliance with professional secrecy.

Cases 6 and 7 refer to patients whose decision-making capacity is unaffected, so they can make decisions about the treatments they are willing to accept. The examination shows that they are temporo-spatial oriented as well as self aware, they answer the questions correctly, understand the objectives of the treatment and the consequences of the refusal. The information contained in the informed consent form for thrombolysis is clear, realistic and at the same time optimistic. The phrasing is simple, uses common terms that do not create confusion. Most of the patients have no problems in understanding and signing informed consent. In some situations, though just asking for consent can make patients/relatives become suspicious and disagree with the proposed treatment or examination. Some maintain their initial refusal, regardless of the additional information that is given to them, as was the situation for the patient presented in the case 6.

In the therapeutic decision-making process, obtaining informed consent enables patients to express their opinion on the benefit-risk ratio for thrombolytic therapy, the effect of thrombolysis and various stroke outcomes. Given that antithrombotic therapy should be given in the utmost urgency, the discussion between the patient or his/her family and the clinician in order to obtain informed consent for thrombolysis may be problematic [32].

Time limits as well as the impact of stroke were the factors that helped design an adequate form in terms of knowledge transmition and content to help making the right decisions in urgent circumstances. The need for a very rapid decision when it comes to rtPA treatment does not allow enough time for reflection. Assimilation of the information is difficult for the patient and family due to the shock of the event and the patients’ cognitive deficits and may generate problems during the decision-making period. In this regard, patients often wanted to let the family decide, but their abilities were also compromised. Emotional and social support has been requested by patients both from family members and from people within their social trust area. The expertise of physicians and health specialists was the basis for making decisions about the treatment with rtPA. The patients and their family described the communication as patronal or paternalist, expecting their views to be respected [33].

In the decision to use i.v. thrombolysis as with any treatment, consent can not be assumed, but the physician’s contribution to decision-making is essential [34]. In the cases presented in this research, that ended with the refusal of the patient, respectively of the legal representative, their decision was a firm one, which did not allow the intervention of the doctor, in order to change it.

In case 7, the patient eventually changes his initial decision to refuse fibrinolytic treatment after a discussion with his daughter; however, the discussion prolonged the time since the stroke was installed until the thrombolysis administration, being well known that the success of the treatment depends on the time elapsed from the onset of the neurological signs to the administration of the treatment. Delayed administration of rtPA has most likely caused haemorrhagic transformation. The doctor warned the patient and his family about this fact. However, this complication may also become the subject of a dispute.

Of the cases presented in our study, the patients who refused the fibrinolytic treatment invoked the fear of hemorrhagic risk. In the hospital where all the cases were registrated, we found a decline rate of approximately 2% over the study period, a smaller percentage compared to those reported by other studies [35]. We have also found that there has been a downward trend in refusal over the last few years. The experience accumulated by physicians over time (both in conducting the thrombolysis procedure and in relation to patients or their legal representatives), the results obtained and the recent scientific evidence that reported t-PA safety for patients with mild symptoms too [36] may influence positively the doctors’ behavior and, implicitly, the increase of patients’ confidence in the benefits of this specific therapy [35]. Also, the recent efforts of the Ministry of Health to raise awareness among the public about the alarming signs of ischemic stroke, showing the benefits of thrombolysis, and the need for treatment may be associated with this decrease.

Some data associate the patient’s refusal with mild symptoms and with the fact that the patient does not realize the severity of the condition, refusing to accept the hemorrhagic risk [32]. There is little data in the literature related to this topic. Standard care refusal was previously looked into from the ethics perspective [37, 38]. From the authors’ point of view, the physicians have the moral responsibility to deal with patients’ refusal and try to discuss it with the patient. Though the patient constantly refuses a treatment, proper and competent efforts should be made to enforce the intervention if the refusal affects the individual or the society. Despite the different opinions about the model of medical care, investigators admit that the role of the physician is not to formulate competence or to take decisions that impair individuals or society using the principle of liberty. The refusal should be studied in all cultural, psychological, behavioral, social and ethical context.

## Conclusions

Medico-legal aspects regarding the administering of rtPA occur when complaints are filed either by the patients or their relatives. These complaints can regard either administering or not the medication. Thorough filling of all medical documents and observing therapeutic guidelines offer protection for attending physicians against such complaints. Obtaining informed consent is currently compulsory and the physician should emphasize not only the benefits of the treatment, which will anyway be noticeable, but rather the complications which may occur. This risk must be taken by the patients or their relatives. However, obtaining informed consent is time consuming. This is especially important in emergency situations, where delaying the fibrinolytic treatment reduces its potential benefits in the recovery of the patient and increases the number of hemorrhagic lesion transformations. It would therefore be extremely useful to simplify consent (without impairing the quality of patient information) and to agree the law in the field with the protocol, given that strokes are major emergencies.

## Data Availability

Any supporting data not provided in the manuscript can be accessed by contacting the authors. All requests should be addressed to Camelia Liana Buhas: cameliabuhas@yahoo.com

## Abbreviations

AF: Atrial fibrillation
ATLANTIS: Alteplase TromboLysis for Acute Noninterventional Therapy in Ischaemic Stroke
CT: Computer tomography
CVA: Cerebral vascular accident
ECASS: European Cooperative Acute Stroke Study
i.a.: Intraarterial
i.v.: Intravenous
IST: International Stroke Trial
NIHS: National Institute of Health Stroke Scale
NINDS: National Institute of Neurological Disorders and Stroke
rtPA: Recombinant tissue plasminogen activator
